# Expanding the food environment framework to include family dynamics: A systematic synthesis of qualitative evidence using HIV as a case study

**DOI:** 10.1016/j.gfs.2024.100788

**Published:** 2024-09

**Authors:** Ramya Ambikapathi, Morgan Boncyk, Nilupa S. Gunaratna, Wafaie Fawzi, Germana Leyna, Suneetha Kadiyala, Crystal L. Patil

**Affiliations:** a Department of Global Development, Cornell University, USA; b Department of Public Health, Purdue University, USA; c Department of Health Promotion, Education and Behavior, University of South Carolina, USA; d Department of Global Health, Harvard Chan School of Public Health, USA; e Department of Epidemiology and Biostatistics, Muhimbili University of Health and Allied Sciencesr, Tanzania; f Tanzania Food and Nutrition Center, Tanzania; g Department of Epidemiology and Population Health, London School of Hygiene & Tropical Medicine, London, UK; h Department of Health Behavior and Biological Sciences, University of Michigan, Ann Arbor, USA

**Keywords:** Qualitative evidence synthesis, Family food environment, Low- and middle-income countries, HIV, Family dynamics, Drivers of food choice

## Abstract

Food environment changes in low- and middle-income countries are increasing diet-related noncommunicable diseases (NCDs). This paper synthesizes the qualitative evidence about how family dynamics shape food choices within the context of HIV (Prospero: CRD42021226283). Guided by structuration theory and food environment framework, we used best-fit framework analysis to develop the Family Dynamics Food Environment Framework (FDF) comprising three interacting dimensions (resources, characteristics, and action orientation). Findings show how the three food environment domains (personal, family, external) interact to affect food choices within families affected by HIV. Given the growing prevalence of noncommunicable and chronic diseases, the FDF can be applied beyond the context of HIV to guide effective and optimal nutritional policies for the whole family.

## Highlights

• Family is an important medium connecting the external and personal food environment (FE) domains.
• Family dynamics enable and/or restrict food choices in HIV-affected households.
• Including family dynamics in the FE is applicable to other diet-related NCDs.
• This family dynamics food environment framework can be used to optimize policies and intervention toward nutritious food choice that is effective for the whole family.

## 1 Introduction

The food environment, where people procure food, shapes food choices, dietary patterns, and nutrition outcomes. Macrolevel factors such as globalization and urbanization shifted food environments toward cheap, convenient, energy-dense, salty, and sugary foods. These factors and associated shifts in food choices create a significant dietary risk for noncommunicable diseases (NCDs) (Delobelle, 2019; Juul et al., 2021; Reardon et al., 2021; Turner et al., 2020; Barrett et al.; Battersby and Watson, 2018). Globally, poor diets are the fifth leading cause of mortality. As such, food environments and choices – how and why people choose foods – have gained considerable attention in policies.

Using Turner's framework, the food environment in low- and middle-income countries can be conceptualized as two major interacting domains, the external and personal, each with describing factors related to food procurement and consumption that drive food choices (Turner et al., 2020; Turner et al., 2018). The external domain includes food availability, prices, vendor and product properties, marketing, and regulations, while the personal domain includes accessibility, affordability, convenience, and desirability. However, this framework does not account for family dynamics.

Expanding the scope of the food environment framework to incorporate family dynamics can offer valuable insights for designing effective family-based interventions and structure policies for optimal family health outcomes, especially among those affected by chronic diseases. Family plays an essential role in managing chronic diseases, especially the family members of people living with Human Immunodeficiency Virus (PLHIV) (Belsey, 2006; Aga et al., 2014; Weiser et al., 2011; Naidu and Harris, 2005; Iwelunmor et al., 2008). Here, family is defined as “any configurations of people who regularly eat together, eat from the same household food resources, and who mutually influence decisions about their family” (Gillespie and Gillespie, 2007). HIV, with improved prevention and treatment, is now considered to be a chronic disease. However, changes in inflammation and fat deposition from treatment make PLHIV more vulnerable to diet-related non-communicable diseases, known as the HIV-related NCDs syndemic (Graff, 2021; Kamkuemah et al., 2021; Popkin, 2006; Patel et al., 2018). Thus, dietary risk factors and the family dynamics affecting food choices, are essential to preventing and managing NCDs.

There are well-established linkages between HIV disease progression, food access, and family support (Belsey, 2006; Aberman et al., 2014). HIV intervention efforts have prioritized food assistance and supplementation interventions alongside HIV treatment because of the bidirectional linkages between disease progression and household food security (Weiser et al., 2011; Anema et al., 2014; Ivers et al., 2009). While the personal and external domains of the food environment are pertinent for households with a PLHIV, accounting for the familial factors shaping the food choices of PLHIV and their family members is needed. We propose integrating a family food environment domain into Turner's framework to show how this domain also shapes the food choices of PLHIV (Turner et al., 2020; Turner et al., 2018; Giddens, 1991; Anthony, 1984).

Using a systematic qualitative evidence synthesis (QES), we aim to demonstrate interactions among the agency of personal food environments and the economic, cultural, religious, and gender structure of the external food environment through the family (Giddens, 1991; Anthony, 1984; Slater et al., 2012; Sobal and Bisogni, 2009). We posit that the family is an important intermediary where structures converge to operationalize the development of habitual food choices and consumption practices. Structural changes will lead to new individual and family routines and rituals and, thus, establish new systems of practices. In the context of a chronic disease diagnosis, such as HIV, the family food environment can (mal)adapt to accommodate or bound food choices and create new food routines (Boncyk et al., 2022).

## 2 Methods

We conducted a qualitative evidence synthesis (QES; Prospero registration: CRD42021226283), a review methodology for rigorous and systematic appraisal and synthesis of qualitative research (Cooke et al., 2012; Flemming and Noyes, 2021). This review aimed to describe and conceptualize the family food environment and explore the family's role in PLHIV food choices, including food acquisition decision-making, preparation, allocation, consumption, and other dietary-related practices (Carroll et al., 2013; Thomas and Harden, 2008). The quality of the articles was evaluated independently by two reviewers using the Critical Appraisals Skills Programme (CASP) tool and confirmed by two different reviewers (Critical Appraisal Skills Programme, 2018).

### 2.1 Search strategy

We searched PubMed, Scopus, and Web of Science with the following keywords and limited word search to qualitative studies filters: “Food and HIV”, “HIV and nutrition”, “HIV and caregiver”, “HIV and family”, “HIV and eating”, “HIV and family”. Two additional searches were conducted, first with a restricted filter “Human, AIDS, Adults” using the following keywords: “Food and Culture”, “Food and Choice”, “Food and consumption”, and “Food and insecurity.” Additionally, we identified 23 review articles during the screening process and searched the references cited in these reviews. Our systematic search yielded 6,783 non-duplicate articles. Two reviewers (RA, MB) independently screened 10% of articles for agreement on title, abstract, and three rounds of full-text screening before independently screening the remaining articles. In the first round of full-text screening, we confirmed the eligibility criteria. In the second round, we identified family-level factors influencing PLHIV food intake and developed a key concepts matrix using grounded theory and *a priori* coding based on Turner's food environment framework (Turner et al., 2020; Turner et al., 2018). Finally, in the third and final rounds, we ensured that included studies contributed to the Family Dynamic Framework. We used the *Colandr* web application to organize the screening process (Kahili-Heede and Hillgren, 2021). This search includes articles published from 1985 to 2020.

### 2.2 Screening

Screening inclusion criteria for articles were as follows: 1) studies conducted in LMIC as defined by the World Bank (2019 definition), 2) qualitative methodology, and 3) content related to HIV and food, including HIV stigma, caregiver burden, food access and availability, food security, food and treatment, food sources, body perception, gender differences/inequality/roles, children caring for HIV parent(s), medication adherence, poverty, disclosure, barriers, basic resources, body image/changes, and sexual transactions. In the second round of full-text screening, we specifically examined how HIV influenced food choices at the family level. The family level was defined as how family or household-level factors affect PLHIV food intake, food acquisition (purchasing, borrowing, production), and food preparation and consumption decision-making. Articles were excluded if the content was on the pediatric HIV population, such as grandparents caring for HIV child orphans and HIV maternal care/breastfeeding. Sixteen articles were excluded because we could not access the full texts.

### 2.3 Data extraction, analysis, and synthesis

Each included study was treated as a transcript. We used the *best-fit framework synthesis* approach to assess and build on Turner's food environment framework (Turner et al., 2020; Turner et al., 2018; Giddens, 1991; Anthony, 1984; Slater et al., 2012). A best-fit framework synthesis is an analytical approach that builds or tests an existing framework (in this case, food environment framework) with new qualitative synthesis like thematic analyses. A family food environment refers to any factors that affect food choice, acquisition, preparation, consumption, or family members' practices related to food choices of PLHIV. We began the analysis with a set of *a priori* themes and codes based on the guiding framework and theory: external, personal, and family food environment. We applied open, axial, and selective coding to identify additional constructs, determine relationships between them, and integrate codes for a deeper understanding of overarching themes. Data not easily accommodated within the framework required iterative interpretation; therefore, we also used inductive analysis techniques to synthesize the data and expand the framework (Suri, 2013). We integrated insights from both the *a priori* codes and emergent constructs to understand the dynamics around food in households affected by HIV.

Data extraction was completed systematically and cross-validated by two authors (RA, MB) and with a weekly discussion of each full-text screened article with the senior author (CP). We extracted the profile information, including the author's name, publication date, study design, and location for each article. First, data were extracted and placed in a matrix based on key concepts. Then they were categorized into personal (body image, food preferences, hunger), family (prioritizing PLHIV, nutrition knowledge, caregiver burden, disclosure, gender difference, financial, social network, food security), and distal (external, food aid, environment) factors and coded in MAXQDA and Excel. Factors such as affordability, accessibility, and convenience were coded as family food environment if they explicitly referred to the family level. Second, given the high prevalence of articles on food security and financial burden and existing literature on HIV and food security (Weiser et al., 2011), we assessed these articles separately to examine how they clustered with the food environment framework. Lastly, after conceptualizing the family food environment domain with three distinct sub-dimensions, the tagged articles on food security experience were re-read and coded guided by the new family domain.

**Fig. 1:**
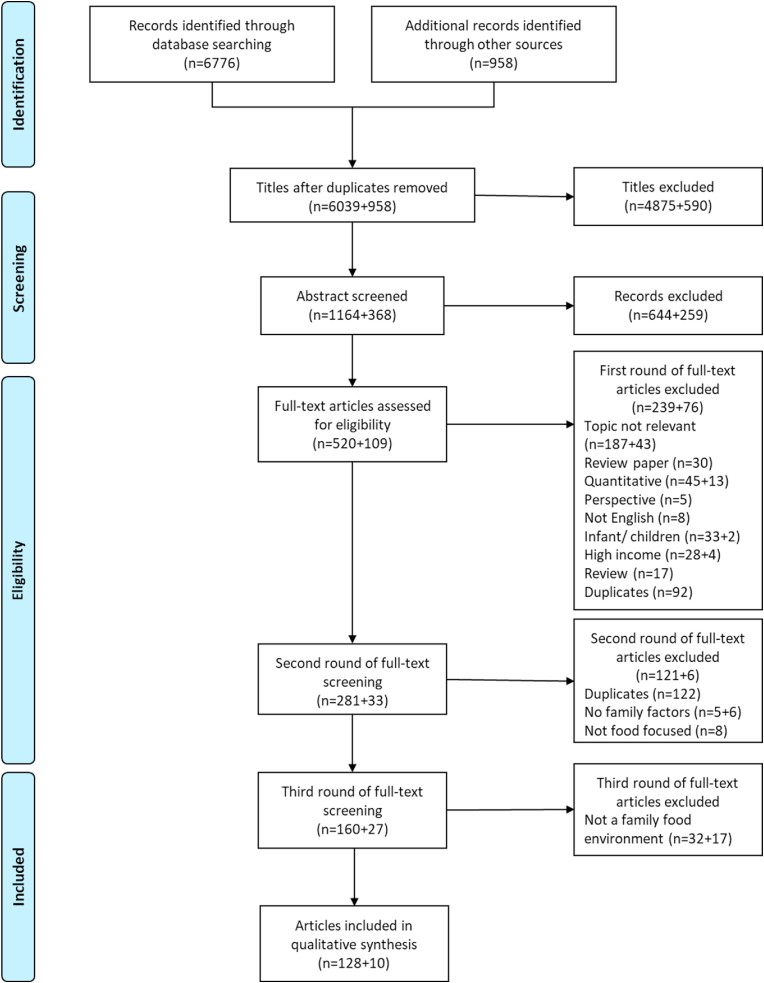
study review process.

After screening three databases and 23 review articles, 6783 articles were included in this review. After title screening, 1532 abstracts were screened. Among those abstracts, 629 articles moved to three rounds of full-text screening (described above). The final review included 138 full texts (Fig. 1). Articles were primarily from Africa (n = 132), with less than 10% from Southeast Asia (n = 10), Latin American (n = 11), or Caribbean (n = 11) regions (Supplemental Figure 1). Publication dates ranged from 1993 to 2020, with 68% of articles published after 2009 (Table 1). Of the 138 included articles, 110 employed structured or in-depth interviews (IDIs), 56 focus group discussions (FGDs), and 61 relied on multiple methods (FGDs, IDIs, observations, case studies, diary entries, photovoice).

**Table 1:** Descriptive characteristics of the included articles and matrix of family food environment identified in the included articles (N = 138).

Study	Study location	Qualitative data type	Resources				Characteristics						Action orientation			Health Context				
			Social capital	Resource allocation	Household wealth	Time use	Composition			Household health status		Household Size	Support	Value negotiations		Impact on livelihoods Livelihoods	Healthcare	Community Support	Acceptance	Nutrition awareness
							Generations	Gender	Aging	Co-morbidities	Chronic Diseases			Competing basic needs	Family desirability					
Aga et al., 2009a	Ethiopia	SSIs, observations			**✓**									**✓**				**✓**		
Aga et al., 2009b	Ethiopia	SSIs, observations					**✓**	**✓**				**✓**	**✓**							**✓**
Aga et al., 2014	Ethiopia	IDIs, observations											**✓**	**✓**		**✓**	**✓**			**✓**
Agbonyitor, 2009	Nigeria	IDIs, FGDs	**✓**				**✓**						**✓**	**✓**		**✓**	**✓**	**✓**		
Alemu et al., 2013	Ethiopia	IDIs, FGDs																	**✓**	
Alomepe et al., 2016	Cameroon	IDIs			**✓**			**✓**					**✓**	**✓**		**✓**		**✓**	**✓**	
Amurwon et al., 2017	Uganda	IDIs	**✓**										**✓**			**✓**		**✓**	**✓**	
Andersen, 2012	Kenya	IDIs, FGDs, observations, drama, diaries	**✓**		**✓**	**✓**	**✓**			**✓**			**✓**	**✓**		**✓**		**✓**		
Aransiola et al., 2014	Nigeria	IDIs	**✓**													**✓**	**✓**	**✓**	**✓**	
Araujo et al., 2018	Brazil	SSIs							**✓**										**✓**	
Asgary et al., 2013, Asgary et al., 2013	Ethiopia	Interviews, FGDs											**✓**						**✓**	
Atukunda et al., 2017	Uganda	IDIs	**✓**										**✓**	**✓**		**✓**				
Atuyambe et al., 2014	Uganda	SSIs						**✓**					**✓**						**✓**	**✓**
Axelsson et al., 2015	Lesotho	IDIs			**✓**	**✓**							**✓**				**✓**		**✓**	
Ayieko et al., 2018	Kenya, Uganda	IDIs				**✓**		**✓**									**✓**		**✓**	
Balaile et al., 2007	Tanzania	IDIs					**✓**	**✓**						**✓**						
Balcha et al., 2011	Ethiopia	SSIs, FGDs															**✓**		**✓**	
Baylies, 2002	Zambia	Interviews	**✓**			**✓**		**✓**					**✓**	**✓**		**✓**	**✓**	**✓**		
Beckett et al., 2016	Haiti	FGDs	**✓**	**✓**	**✓**												**✓**	**✓**	**✓**	
Bezabhe et al., 2014	Ethiopia	SSIs, FGDs	**✓**				**✓**						**✓**			**✓**	**✓**	**✓**	**✓**	
Bindura-Mutangadura, 2001	Zimbabwe	IDIs, FGDs	**✓**	**✓**			**✓**	**✓**					**✓**	**✓**	**✓**		**✓**	**✓**		
Braathen et al., 2016	Malawi	IDIs, observations (home, clinic), case study		**✓**		**✓**				**✓**			**✓**				**✓**		**✓**	**✓**
Burgess and Campbell, 2014	South Africa	IDIs	**✓**		**✓**		**✓**	**✓**					**✓**	**✓**		**✓**		**✓**		
Byron et al., 2008	Kenya	IDIs, FGDs	**✓**	**✓**								**✓**		**✓**		**✓**	**✓**	**✓**		
Campbell et al., 2011	Zimbabwe	SSIs, FGDs	**✓**				**✓**									**✓**		**✓**	**✓**	
Chazan, 2014	South Africa	IDIs, FGDs		**✓**	**✓**		**✓**	**✓**	**✓**	**✓**		**✓**	**✓**	**✓**		**✓**	**✓**	**✓**	**✓**	
Conroy et al., 2018	Malawi	IDIs		**✓**			**✓**	**✓**					**✓**			**✓**	**✓**			
Crane et al., 2006	Uganda	IDIs		**✓**							**✓**		**✓**	**✓**			**✓**			
Czaicki et al., 2017	Tanzania	IDIs		**✓**		**✓**	**✓**			**✓**				**✓**		**✓**	**✓**			
deGraft et al., 2002	Zimbabwe	SSIs, observations			**✓**		**✓**						**✓**	**✓**			**✓**			
Derose et al., 2017	Dominican Republic	IDIs	**✓**	**✓**			**✓**						**✓**			**✓**		**✓**	**✓**	
Dinh et al., 2018	Vietnam	Interviews		**✓**			**✓**				**✓**		**✓**						**✓**	
Dovel and Thomson, 2016	Uganda	IDIs		**✓**	**✓**	**✓**	**✓**	**✓**				**✓**								
Du and Lekganyane, 2010	South Africa	Group IDIs, observations	**✓**	**✓**	**✓**	**✓**		**✓**								**✓**		**✓**		
Dworkin et al., 2013	Kenya	IDIs											**✓**			**✓**				
Fielding-Miller et al., 2014	Swaziland	IDIs					**✓**	**✓**					**✓**			**✓**			**✓**	
Gebremariam et al., 2010	Ethiopia	IDIs, FGDs											**✓**							
Gombachika et al., 2014	Malawi	IDIs					**✓**	**✓**					**✓**	**✓**		**✓**				
Goudge and Ngoma, 2011	South Africa	IDIs, FGDs	**✓**	**✓**									**✓**			**✓**	**✓**	**✓**	**✓**	**✓**
Gwatirisa and Manderson, 2009	Zimbabwe	IDIs, FGDs, observations	**✓**	**✓**	**✓**						**✓**		**✓**	**✓**		**✓**		**✓**		**✓**
Hardon et al., 2007	Botswana, Tanzania, Uganda	SSIs, FGDs					**✓**						**✓**							**✓**
Hatcher et al., 2020	Kenya	IDIs		**✓**	**✓**		**✓**			**✓**			**✓**	**✓**		**✓**				
Herce et al., 2014	Malawi	SSIs	**✓**			**✓**							**✓**			**✓**	**✓**	**✓**		
Holzemer et al., 2007	Lesotho, Malawi, South Africa, Swaziland, Tanzania	FGDs											**✓**							
Horn and Brysiewicz, 2014	South Africa	Interviews															**✓**			
Hussen et al., 2014	Ethiopia	IDIs, observations, photovoice sessions, group discussion	**✓**										**✓**					**✓**	**✓**	
Iwelunmor et al., 2008	South Africa	FGDs	**✓**										**✓**					**✓**	**✓**	
Jones et al., 2009	Zambia	FGDs											✓							
Jones, 2011	South Africa	SSIs, interviews informal, observations											✓	✓			✓	✓		✓
Kaler et al., 2010	Uganda	Interviews	**✓**	**✓**	**✓**		**✓**						**✓**	**✓**	**✓**	**✓**	**✓**			
Kalofonos, 2010	Mozambique	SSIs, observations		**✓**								**✓**						**✓**		
Kang'ethe, 2009a	Botswana	Interviews, FGDs	**✓**		**✓**	**✓**	**✓**	**✓**					**✓**			**✓**		**✓**		**✓**
Kang'ethe, 2009b	Botswana	IDIs, FGDs		**✓**			**✓**									**✓**				
Kebede and Haidar, 2014	Ethiopia	FGDs		**✓**			**✓**						**✓**		**✓**					
Kellett and Gnauck, 2017	Uganda	Interviews, FGDs		**✓**			**✓**	**✓**								**✓**				
King et al., 2018	South Africa	SSIs, observations (clinic)			**✓**		**✓**						**✓**			**✓**				
Kipp et al., 2007	Uganda	IDIs	**✓**			**✓**	**✓**	**✓**					**✓**	**✓**		**✓**		**✓**		
Klunklin and Greenwood, 2005	Thailand	Interviews, observations (home)					**✓**	**✓**					**✓**							
Knight et al., 2016	South Africa	SSIs, observations (home)	**✓**				**✓**	**✓**					**✓**			**✓**		**✓**		
Kohli et al., 2012	India	IDIs, FGDs	**✓**		**✓**	**✓**	**✓**	**✓**					**✓**					**✓**		**✓**
Kuteesa et al., 2012	Uganda	IDIs, FGDs, observations (clinic)							**✓**				**✓**						**✓**	
Laker and Ssekiboobo, 2003	Uganda	FGDs	**✓**		**✓**	**✓**	**✓**	**✓**						**✓**	**✓**	**✓**				**✓**
Li et al., 2008	China	IDIs											**✓**						**✓**	
Linda, 2013	South Africa	IDIs, interviews informal, FGDs, observations (home)	**✓**				**✓**			**✓**	**✓**		**✓**			**✓**		**✓**	**✓**	
Majumdar and Mazaleni, 2010	South Africa	IDIs, FGDs		**✓**			**✓**							**✓**		**✓**				
Makoae, 2011	Lesotho	IDIs			**✓**								**✓**							**✓**
Mangesho, 2011	Tanzania	Interviews formal and informal, group discussions, observations (meetings, clinic)	**✓**	**✓**			**✓**	**✓**		**✓**			**✓**	**✓**	**✓**	**✓**		**✓**		**✓**
Martin et al., 2011	Latin America and Caribbean	SSIs	**✓**			**✓**							**✓**	**✓**			**✓**	**✓**		
Maughan-Brown et al., 2019	South Africa	FGDs				**✓**								**✓**			**✓**			
Mendelsohn et al., 2014	Kenya, Malaysia	IDIs	**✓**										**✓**			**✓**	**✓**	**✓**	**✓**	
Mill and Anarfi, 2002	Ghana	IDIs, FGDs	**✓**		**✓**		**✓**	**✓**			**✓**		**✓**	**✓**		**✓**	**✓**			
Miller and Tsoka, 2012	Malawi	SSIs					✓			✓			✓	✓						
Miller et al., 2011	Uganda	IDIs		✓			✓	✓					✓			✓			✓	
Mkandawire et al., 2015	Malawi	IDIs, FGDs		**✓**	**✓**			**✓**					**✓**	**✓**				**✓**		
Mkandawire-Valhmu et al., 2013	Kenya, Malawi	SSIs, FGDs			**✓**		**✓**	**✓**								**✓**	**✓**			
Mooney et al., 2017	South Africa	IDIs	**✓**															**✓**	**✓**	**✓**
Moore and Williamson, 2003	Togo	Interviews	**✓**														**✓**	**✓**		
Moyo et al., 2017	Zimbabwe	IDIs		**✓**	**✓**		**✓**	**✓**					**✓**			**✓**				
Mshana et al., 2006	Tanzania	IDIs, FGDs		**✓**		**✓**							**✓**				**✓**	**✓**	**✓**	
Mukumbang et al., 2017	Zambia	IDIs, FGDs				**✓**											**✓**		**✓**	
Musumari et al., 2013	Democratic Republic of Congo	IDIs		**✓**	**✓**									**✓**						
Nachega et al., 2006	South Africa	IDIs, FGDs	**✓**										**✓**				**✓**	**✓**		
Nagata et al., 2012	Kenya	SSIs					**✓**	**✓**								**✓**				
Naidu and Sliep, 2012	South Africa	Interviews unstructured						**✓**					**✓**					**✓**		
Nam et al., 2008	Botswana	IDIs														**✓**				
Nankwanga et al., 2009	Uganda	IDIs, FGDs	**✓**				**✓**						**✓**				**✓**	**✓**		
Ngamvithayapong-Yanai et al., 2005	Thailand	IDIs, observations (home)	**✓**				**✓**	**✓**		**✓**			**✓**			**✓**			**✓**	**✓**
Nkosi et al., 2006	Democratic Republic of Congo	FGDs		**✓**	**✓**						**✓**		**✓**			**✓**			**✓**	
Nsimba et al., 2010	Tanzania	SSIs, FGDs, observations											**✓**						**✓**	
Ogunmefun and Schatz, 2009	South Africa	IDIs	**✓**	**✓**	**✓**	**✓**	**✓**	**✓**					**✓**	**✓**		**✓**	**✓**	**✓**		
Okoror et al., 2013	Nigeria	IDIs											**✓**			**✓**	**✓**		**✓**	
Olenja, 1999	Kenya	Interviews, FGDs			**✓**			**✓**					**✓**					**✓**	**✓**	
Olsen et al., 2013a	Ethiopia	IDIs, observations		✓			✓				✓						✓		✓	
Olsen et al., 2013b	Ethiopia	Interviews informal, FGDs, observations (home)	✓														✓		✓	
Oluwagbemiga, 2007	Nigeria	IDIs, FGDs		**✓**					**✓**					**✓**			**✓**			
Orner, 2006	South Africa	IDIs	**✓**			**✓**		**✓**						**✓**				**✓**		
Palar et al., 2013	Bolivia	SSIs						**✓**								**✓**				
Pallangyo and Mayers, 2009	Tanzania	SSIs		**✓**	**✓**	**✓**	**✓**	**✓**				**✓**	**✓**	**✓**		**✓**	**✓**			
Parker et al., 2009	Uganda	SSIs			**✓**	**✓**	**✓**	**✓**					**✓**			**✓**	**✓**	**✓**		**✓**
Paz-Soldán et al., 2013	Peru	IDIs								**✓**			**✓**							
Raniga and Simpson, 2010	South Africa	SSIs	**✓**				**✓**		**✓**		**✓**	**✓**	**✓**	**✓**		**✓**	**✓**	**✓**		
Rodas-Moya et al., 2016	Malawi	IDIs		**✓**			**✓**			**✓**		**✓**	**✓**				**✓**			**✓**
Rodas-Moya et al., 2017	Thailand	IDIs																	**✓**	
Rödlach, 2009	Zimbabwe	SSIs, FGDs, observations (home)	**✓**										**✓**				**✓**	**✓**		
Root, 2010	Swaziland	SSIs						**✓**					**✓**							
Rowe et al., 2005	South Africa	IDIs		**✓**				**✓**					**✓**	**✓**			**✓**			
Russell et al., 2016	Uganda	IDIs		**✓**								**✓**		**✓**		**✓**				
Salter et al., 2010	Vietnam	IDIs		**✓**									**✓**						**✓**	
Samuels and Rutenberg, 2011	Kenya, Zambia	IDIs, FGDs	**✓**	**✓**									**✓**			**✓**		**✓**		
Sanjobo et al., 2008	Zambia	IDIs, FGDs											**✓**						**✓**	
Schatz, 2007	South Africa	IDIs	**✓**	**✓**		**✓**	**✓**	**✓**	**✓**				**✓**	**✓**		**✓**		**✓**		
Schatz and Gilbert, 2012	South Africa	SSIs	**✓**		**✓**				**✓**	**✓**			**✓**	**✓**			**✓**	**✓**		
Schatz et al., 2011	South Africa	SSIs	**✓**				**✓**	**✓**	**✓**			**✓**	**✓**	**✓**		**✓**	**✓**			
Schatz et al., 2019	Uganda	IDIs			**✓**	**✓**			**✓**	**✓**			**✓**				**✓**		**✓**	**✓**
Scott et al., 2014	Zimbabwe	Interviews, FGDs, observations	**✓**	**✓**				**✓**					**✓**				**✓**	**✓**	**✓**	
Seeley et al., 1993	Uganda	Interviews informal, observations	**✓**				**✓**	**✓**					**✓**				**✓**			
Selman et al., 2013	Kenya, Uganda	IDIs	**✓**													**✓**	**✓**		**✓**	
Sileo et al., 2016	Uganda	FGDs														**✓**				
Sisya, 2010	Zambia	IDIs, FGDs	**✓**										**✓**				**✓**	**✓**	**✓**	
Ssengonzi, 2007	Uganda	IDIs, FGDs					**✓**	**✓**	**✓**				**✓**							
Tanyi et al., 2018	Cameroon	IDIs, FGDs	**✓**																	
Thomas, 2006	Namibia	Diaries	**✓**	**✓**	**✓**	**✓**	**✓**	**✓**		**✓**	**✓**		**✓**	**✓**		**✓**				
Tshililo and Davhana-Maselesele, 2009	South Africa	IDIs		**✓**										**✓**		**✓**				
Tuller et al., 2010	Uganda	IDIs			**✓**									**✓**			**✓**			
VanTyler and Sheilds, 2015	Kenya	IDIs					**✓**	**✓**					**✓**	**✓**		**✓**				
Wacharasin and Homchampa, 2008	Thailand	IDIs, FGDs, obervations (home, clinic)											**✓**			**✓**			**✓**	**✓**
Ware et al., 2009	Nigeria, Tanzania, Uganda	IDIs, observations (clinic)	**✓**	**✓**									**✓**	**✓**			**✓**	**✓**		
Watt et al., 2009	Tanzania	IDIs					**✓**	**✓**					**✓**				**✓**		**✓**	
Webel et al., 2017	Botswana	FGDs								**✓**				**✓**						
Weiser et al., 2010	Uganda	SSIs		**✓**			**✓**	**✓**		**✓**				**✓**			**✓**	**✓**		
Weiser et al., 2017	Kenya	IDIs					**✓**						**✓**	**✓**		**✓**	**✓**		**✓**	**✓**
Williams and McGill, 2011	Mozambique	IDIs, FGDs			**✓**			**✓**								**✓**		**✓**		
Wright et al., 2012	Uganda	SSIs	**✓**				**✓**		**✓**	**✓**			**✓**	**✓**		**✓**				
Xie et al., 2017	China	IDIs											**✓**						**✓**	
Yager et al., 2011	Uganda	IDIs						**✓**								**✓**		**✓**		
Yakob and Ncama, 2016	Ethiopia	IDIs, FGDs, case study		**✓**	**✓**		**✓**						**✓**	**✓**		**✓**				**✓**
Yizengaw et al., 2013	Ethiopia	Interviews, FGDs	**✓**	**✓**	**✓**								**✓**			**✓**				
Zembe et al., 2013	South Africa	Interviews, FGDs			**✓**									**✓**		**✓**				
IDIs = in-depth interviews; FGDs = focus group discussions; SSIs = semi-structured interviews																				

## 3 Results

Nearly all articles used appropriate qualitative methodology (98%) and explicitly stated the research aim of the study (96%). Most articles adequately detail participant recruitment (92%) and data collection (98%). A fifth (19%) of articles did not consider the relationship between the researcher and participants, and a third (32%) did not indicate ethical consideration. Quality assessments of the articles are summarized in Supplemental Table 1.

Family Food Environment Domain.

**Fig. 2:**
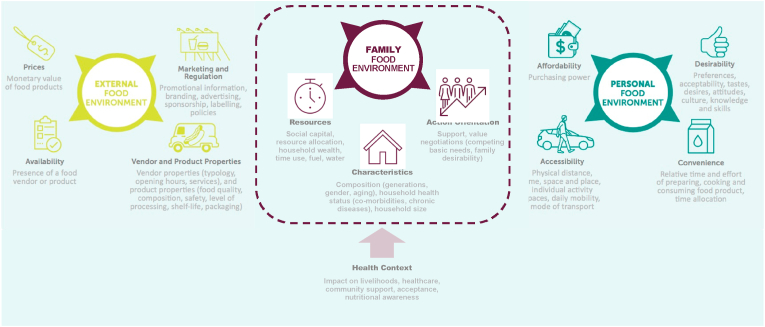
The Family Dynamics Framework (FDF) is theoretically informed (Giddens, 1991; Anthony, 1984) and expands existing frameworks (Turner et al., 2020; Turner et al., 2018) to show the additional family food environment domain and associated dimensions related to drivers of food choice in the context of families affected by HIV.

We hypothesized that the family food environment domain would be an intermediary between Turner's external and personal food environment domains (Turner et al., 2020; Turner et al., 2018). This family domain captures how the external food environments, rules, and rituals, also termed structures, bind and expand agency to affect the food choices of the personal food environment (Turner et al., 2020; Giddens, 1991). From the synthesis of 138 articles, we expanded the original framework to derive the Family Dynamics Framework (FDF) (Turner et al., 2018). FDF is characterized by: 1) resources available, 2) family characteristics, and 3) the action orientation of the family, which occurs within 4) a health context (Fig. 2). Each of the three dimensions includes several factors (defined in Table 2) that function independently and interact to influence family-level food choices that affect individual food choices within a household. Results are organized by how the family domain functions to enable and bind choices along with a summary of illustrative quotes and references for each factor (Table 3).

**Table 2:** Definition of the family food environment domain, dimension, factor, sub-factor within the Family Dynamics Framework (FDF). Family food environment domain: essential intermediary between external and internal food environment that captures how the structures (external food environments, rules, and rituals) bound and expand agency intersection to affect the food choices of the personal food environment.

**Dimension**	**Factor**	**Sub-factor**
**Resources**: Pooled materials and resources related to food acquisition and preparation that affect food choices	**Social capital:** Social network, support, and trust that bond, bridge, and link PLHIV and their family with a network (neighbors, extended family, work) and affects preferred food allocation toward a PLHIV	
	**Resource allocation:** How households pool, divide and distribute food quantity and quality	
	**Household wealth:** Financial capital and assets available within a household	
	**Time use:** Time lost when PLHIV no longer participates in labor and household chores as well as the time that a family member spends on caring for the PLHIV	
**Characteristics**: Composition that affects resources and decision-making regarding food preferences and how these factors affect PLHIV and family member's food choices and consumption patterns	**Composition:** Family members of different ages, generations, and genders residing in the same complex, whether under the same roof, within a shared compound, or in adjacent dwellings, influencing the dynamic of food choice	**Generations:** How multigenerational, extended, female- or male-headed households and children impacted food choices
		**Gender:** Social roles ascribed to men and women impact food choice
		**Aging:** Increased health risks and additional support that PLHIV need later in life
	**Household Health Status:** Disease navigation or how the family and PLHIV make food decisions	**Co-morbidities**: Co-occurring morbidities besides their HIV diagnosis that require additional care and a tailored diet
		**Chronic diseases**: Morbidities among other family members
	**Household Size:** Number of family members affecting the food choices of the PLHIV and household members	
**Action orientation:** Strategies and observable acts affecting food allocation decisions and diets of PLHIV	**Support:** Family factors that enable food choices of PLHIV and their family members	
	**Value negotiations:** Factors that compete with individual preferences within the family	**Competing basic needs:** Prioritizing one family member over another when household resources are scarce, thereby impacting the well-being of family members
		**Family desirability:** Balancing all family food preferences and needs while accounting for norms related to religion, ethnicity, culture, or region
**Health Context:** How the FDF fits within the chronic disease of focus	**Impact on livelihoods:** Lost income due to disease management of an individual with a chronic disease and their family members	
	**Healthcare:** Burden associated with disease treatment, including hidden costs such as clinic transportation, waiting time, and testing, and how these healthcare burdens impact food choices	
	**Community support:** Structural networks, hospital, and organized community groups an individual with a chronic disease can rely on to support their food choices	
	**Acceptance:** How the household's awareness of the chronic disease status and their demonstration of acceptance through the levels of support they provide	
	**Nutritional awareness:** How the family domain worked to optimize the personal food environment by enabling healthier food choices for an individual with a chronic disease when family members are aware of the person's nutritional needs

**Table 3:** Supportive quotes of subdimensions of the Family Dynamics Framework (FDF).

1. RESOURCES
*Social capital*
1.1a “My children after seeing the state I was in and after getting ARVs [ART], … They got encouraged and as a result they buy me passion fruits and sugar.” (Hardon et al., 2007)
1.1b “… previously, they [PLHIV's parents] kept the food to themselves, but since they learned about my HIV, they reserved or shared the tasty food with me by bringing it to my house.” (Salter et al., 2010)
1.1c “… a treatment partner describes how ‘having friends who have shops’ provides access to credit that enables her to accommodate the food preferences of the patient she helps, who is not doing well: ‘The patient is seriously sick now. … we are borrowing rice from people with shops. They trust us and they lend, paying is a problem. I live with good neighbors who have shops.’” (Ware et al., 2009)
*Resource allocation*
1.2a “We know my mother [who is affected by HIV] needs to eat because she is sick so sometimes when she gets money from her ‘genge’ she can go and buy food for herself when we also have something to eat. We know she is sick so we cannot force her always to bring money for us.” (Mangesho, 2011)
1.2b “It was described as especially difficult not to share [food supplements] with children. A participant told her husband not to eat RUSF, but she said that telling a child would have been impossible: … If it were little children, I would be forced to give.” (Olsen et al., 2013a)
*Household wealth*
1.3 “‘ … In this community we have tended to think that the western foods represent being advanced. So when I get money for groceries, I tend to buy the refined foods partly because they characterise our consumption patterns although they are not healthy’ (IDI, male, 38 years). However, the respondents indicated that they relied mostly on wild fruits, as they could not afford to buy the exotic fruits sold in the supermarkets. All the participants indicated that during tough economic times they relied mostly on wild vegetables they could gather from the field such as mushroom and wild plants. They felt that the wild vegetables were rich in proteins and highly nutritious.” (Moyo et al., 2017)
*Time use*
1.4 “Wealthier families could hire labour to replace the caregiver's time, but for most families, AIDS meant the loss of two workers, not one … [as an HIV caregiver explained]. For almost a month, I did not go to the garden. I stayed at home taking care of him, cooking and washing.” (Kaler et al., 2010)

2. CHARACTERISTICS
Composition
Generations
2.1a “[I] went back to my hometown to live with my parents when my husband died. … they look after my son. My mum cooks for me. They also give me many supports. … [and] money to buy medicines.” (Klunklin and Greenwood, 2005)
2.1b “A 57-year-old woman, who was responsible for feeding three grandchildren as well as her two youngest children, explained: ‘I eat less food so my children can eat, because their lives are ahead of them, and mine is about to end, and they feel the privation of hunger more than I do. So I eat less.’” (Weiser et al., 2010)
Gender
2.2 “Few husbands regularly helped women in the tasks of cooking, fetching water, washing clothes and utensils; more men regularly helped with taking family members to the doctor, purchasing groceries and providing childcare. There were, however, some men who contributed to the household.” (Davis and Kostick, 2018)
Aging
2.3a “Many of the participants … had lost supportive children and grandchildren or were worried about losing their children as a result of the HIV epidemic. A few were reliant on care and support provided by grandchildren for whom they were responsible, resulting in different levels of mutual responsibility, support and care. Some of the older people received support from their own children, now adults, and some were reliant upon neighbors to help them get food and water …” (Wright et al., 2012)
2.3b “… the elderly modified their lifestyle and their behavior after the HIV/AIDS diagnosis. This triggered changes in the social and health dimensions, causing isolation and reduction of contact with people. … Interruptions of activities, previously routine, may be justified by the embarrassment generated by the diagnosis of an infectious disease, [and] fear that its condition is discovered …” (Araujo et al., 2018)
Household Health Status
Co-morbidities
2.4 “The patients, the shared, may have not only multiple health concerns but also socioeconomic barriers that can impede the patient's ability to engage in self management behaviors. One provider shared about unhealthy eating habits among her patients, ‘[My patients] are eating what is available, that's why [they] get diabetes [and] high blood pressure, because most of … [their eating habits] are not changed’ (Provider, Botswana). While the providers and healthcare team members shared the actions they took to encourage diet and exercise in their interactions with patients, they also recognized that they must also consider the competing needs their patients experienced … One provider shared: … ‘Some patients will tell you that they can't adhere because they don't have anything, no food, nothing …. A home probably trumps (worrying about) the cholesterol.’” (Webel et al., 2017)
Chronic diseases
2.5a “Many participants reported that receiving the incentives reduced stress, worry, and depression, and fostered a sense of peace because they were able to meet basic needs. These results suggest that mental health may have improved temporarily among transfer recipients, although this topic was not included in the interview guide.” (Czaicki et al., 2017)
2.5b “Participants in our study perceived that mental health was altered through several key mechanisms, including: improved food security and ability to provide for family, more productive daily routines (thereby reducing time for and attention to persistent fears), enhanced social standing that accompanied being more active community members.” (Hatcher et al., 2020)
2.5c “… I have to cook soft foods like banana and soup, which she [PLHIV] can eat. I also have to cook for other family members. My parents are old. They can't do anything; they depend on me. I pay attention to the patient because she may need my help… At the end of the day, I find myself exhausted; the day ends just like that.” (Pallangyo and Mayers, 2009)
Household size
2.6 “My health has changed but my diet has not. There are 14 people in my house who need to eat. I appreciate the food I receive, but it's not enough.” (Kalofonos, 2010)

3. ACTION ORIENTATION
Support
3.1a “Since the nausea and fatigue associated with the medication tended to make patients lose their appetite, family members countered this by cooking for them or making their favorite dishes.” (Paz-Soldán et al., 2013)
3.1b “My brother's wife discriminates against me all the times. She says I must go to the person who gave me HIV. At times she cooks food late beyond the time I am supposed to be taking my medication. The living conditions are very difficult for me now, as l don't have a job to enable me to be independent. … [she] has the final say on what she buys. She usually spends the money on herself and sometimes she doesn't buy enough food for the whole month.” (Moyo et al., 2017)
3.1c “We don't have fear of HIV. We will not get [HIV] by touching him; that's why we help. When we go in field area, if such person is there, we sit beside him, eat in the same plate”. But the practices of an ART naïve widow (CS13) were as follows: ‘Now if I ask her [daughter] for a glass of water, then I don't let her drink from the same glass. I don't let her touch it at all. The water that I've drunk, I do not allow anybody to have it. Now I do not share the food from my plate with anybody else at home.’” (Kohli et al., 2012)
3.1d “A secondary driver of food insecurity were disruptions in social networks (family, friends, neighbors) due to HIV-related stigma, which distanced people from important social sources of food support.” (Derose et al., 2017)
Value negotiations
Competing basic needs
3.2a “Children need to eat, the house rent needs to be paid; children fall sick like any other children in the world and therefore need medical treatment. If the business is small, the life becomes very difficult. If you have rented the house, the owner doesn't care that you are sick. If you don't have money for the house, the owner can just take your properties out because really, she/he needs money.” (Pallangyo and Mayers, 2009)
3.2b “… I have been having some financial challenges ever since I lost my job, which she does not seem to understand. Town life is very difficult. We buy everything; food, fees, rent and others … I know its my responsibility to provide for her but we only could afford one small meal a day. She was unhappy with me when it became difficult for me to provide for her special meals, shelter, transport and other needs when I have no job at all so she left to stay with another relative. (study participant's paternal aunt).” (Atukunda et al., 2017)
3.2c “Under such circumstances, there were times when the carer was forced to prioritise immediate household requirements over those of caring, particularly if without assets, the household was unable to generate cash to pay agricultural labourers or to purchase food. The difficulties facing the household therefore have significant repercussions for the well-being of the patient since the carer has far less time and resources to spend ensuring that even the basic needs of the patient are met.” (Thomas, 2006)
Family desirability
3.3 “During Ramadan, I only take the evening [ART] dose. It is impossible to take the morning dose as we eat during the nighttime.” (Bezabhe et al., 2014)

4. HEALTH CONTEXT
Impact on livelihoods
4.1a “The loss of income from patients who were the main breadwinners resulted in severe financial constraints. Household economic problems often began when patients began to suffer from frequent HIV-related illnesses, especially when caregivers were also unable to work because of caregiving responsibilities, thus further reducing financial security.” (Pallangyo and Mayers, 2009)
4.1b “When a husband or adult child falls ill, the older woman often takes over the physical responsibilities and day-to-day tasks of caregiving. … When caregiving takes precedence, other income-generating or resource-gathering tasks may suffer. Some of the respondents talked about such disadvantages. Pearl, a 74-year-old widow, said: The disadvantage is this, you always work hard and you don't get a chance to do your own things. For instance, my husband fell sick during the summer season. I was supposed to go to the field and plough mealies and vegetables but I didn't because I was busy taking care of my husband, so my heart was painful when I saw other women harvesting food ploughed with their own hands.” (Ogunmefun and Schatz, 2009)
4.1c “Days revolve around being able to find adequate resources for their family (food, shelter), and access to school for children. Only one woman had a steady job, which was keeping house 3 days a week for another woman who lived outside Kibera. Others earn money by doing casual work such as washing, braiding hair, making and selling soap, custom crocheting, and catering at community functions when invited.” (VanTyler and Sheilds, 2015)
4.1d “We try working for piece work [*ganyu*] for food or money; however, with the medications [ART] we are on, it is even difficult for us to work for long hours.” (Gombachika et al., 2014)
4.1e “Availability of time to visit the clinic was another major factor that reportedly delayed ART initiation. For many, work commitments and the fear of losing their jobs as a result of the many days required at the clinic in order to start treatment delayed linkage to care or resulted in patients not completing the ART initiation process: ‘ … we have these jobs that we are doing and it's not that easy to stay or ask for days off every week, because they would have a concern about abusing sick leave.’” (Gombachika et al., 2014)
Healthcare
4.2 “Others think that ART is free. They don't see costs associated with the treatment. In fact, we found out that ART was rather expensive. We have to pay for laboratory investigations except CD4 count. We have to pay for other medicines. We pay our transportation fees. Some of us have to stay a night or two. Accommodation is expensive. In general, the town is expensive. And some of us are self- employed. We leave our work for two to three days. These were major reasons for some patients to stop treatment.” (Balcha et al., 2011)
Community support
4.3 “She lived there for two years, then returned to Addis Ababa and eked out a living selling injera; most of her patrons were friends. She continued to get weaker, and a friend who knew her status convinced her to go to ALERT, where physicians started her on ART. Several years later, she met an HIV-positive man at church; they are now married, and she describes her husband as a supportive partner. Both of them are very active in the community and especially in the community coffee ceremony programme. The traditional coffee ceremony is a classic feature of traditional Ethiopian home and community life. The coffee ceremony is a gathering given by village dwellers. We call both those who are HIV positive and negative people and teach them about HIV. We usually get some people who ask for forgiveness for their wrong discriminatory actions they committed, after they understand about problem. I believe all these things happen due to low levels of understanding. That is what the coffee ceremony has brought for us. The community gathers and discusses it openly. The other benefit of coffee ceremony is it provides ways for us [PLHIV] to help each other. For example, if a person is in short of money even to come to ALERT, they will be given some money from the contributions we collect from the crowd at the coffee ceremony. When someone is found ill, all of us will go and visit him/her turn by turn. We also have a saving scheme and we save 10 birr per month in addition to the contribution to coffee ceremony group, which is 2 birr per month. Then we also give a credit service to the members to get a small loan, work with it and pay back with small interest.” (Hussen et al., 2014)
Acceptance
3.4b “Disclosure could be associated with improved access to HIV-care services and therefore earlier presentation: ‘I suffered for two years. Time came when I lost appetite and could not eat food; I weighed 25 kg … My daughter urged me to take an HIV test at a nearby clinic. I disclosed my status to my daughter, but she could not afford my care at [that] clinic. She then brought me here at the Uganda Cares clinic.’” (Kuteesa et al., 2012)
4.4b “The reactions of my family members has not changed since they know my HIV status, in fact, my husband still uses the same plates, cutleries and every other things with me, even when I try to stop him, he is not bothered at all and he is HIV-negative.” (Aransiola et al., 2014)
4.4c “The Zambian practice of shared bowls, utensils and the use of hands to eat lead some participants to express distress at changes in eating arrangements, such as ‘[They] don't want to eat together thinking they will be infected, sometimes they want to use separate kitchen utensils. When you are eating, then a child comes to eat from your plate; he is told not to eat with you.’” (Jones et al., 2009)
4.4d “… [stigmatizing behaviors] included isolation of eating, eating utensils (e.g., AIDS cup), and dishwashing sets, as well as restriction from some food items, (e.g. beef, catfish, egg, preserved foods) or supplementing the diet with special foods or drinks (e.g., milk, nutritional tonic, boiled water). The duration and the degree of Adherent and Nonadherent behaviors were determined partly by HIV status and its stigma.” (Ngamvithayapong-Yanai et al., 2005)
Nutritional awareness
4.5a “Primary family caregivers encouraged healthy nutrition and deemphasized taboo food because they believed that healthy food would increase immune function for the PLWH. A 44-year-old mother and caregiver said the following: ‘I do not allow him to eat pickled food, stingray fish, anchovy fish, or raw food, since he got skin itching and rash after having these foods. No alcohol, since alcohol will react with antivirus drug …. I encouraged him to have organic vegetable and fruit.’” (Wacharasin and Homchampa, 2008)
4.5b “Sometimes she [daughter who is affected by HIV] is in a bad condition and she chooses food that her heart needs. But myself I am poor and I cannot give her what she wants, and sometimes she spends the whole day without eating because I cannot afford what she wants to eat.” (Thomas, 2006)
4.5c “In general, families … are well aware of the links between HIV/AIDS and nutrition, but they are unable to prepare special meals because of the competing demands on their limited financial resources and time. … some special meals had been provided to the sick in the past, when resources were more abundant, but now sick people were eating what was prepared for the whole household.” (Laker and Ssekiboobo, 2003)

### 3.1 Resources is the pooled materials and resources related to food acquisition and preparation that affect food choices, including social capital, resource allocation, household wealth, and time use

**Social capital** refers to the social network, support, and trust (Ferlander, 2007) that bond, bridge, and link PLHIV and their family with a network (neighbors, extended family, work) and affects preferred food allocation toward a PLHIV (38% of articles). Family members within the household and extended family both contribute to and benefit from this social capital. Food was a common medium for operationalizing social capital. To enable the food choices of PLHIV, PLHIV and their families often described reliance on social networks such as extended family members, including adult children living outside the home, and those built through social relationships, such as neighbors and friends (Table 3: 1.1a-b) (Hardon et al., 2007). Adult children of PLHIV living outside the household provided food or money for their HIV-related needs. Prior social relationships with food vendors and neighbors allowed PLHIV to borrow from vendors when necessary (Table 3: 1.1c) (Ware et al., 2009).

**Resource allocation** refers to how households pool, divide, and distribute food quantity and quality (33% of articles). In low-income settings, food allocation decisions were based on energy expenditure, gender, household composition, and competing family needs. Five articles reported that family members prioritized higher food quality for PLHIV without expecting that they would share it with others (Table 3: 1.2a) (Mangesho, 2011). PLHIV found it hard not to share with other family members, especially children (Table 3: 1.2b) (Moyo et al., 2017; Olsen et al., 2013a; Kebede and Haidar, 2014; Czaicki et al., 2017). One study reported variability in who was prioritized by workload seasonality (Mangesho, 2011); larger meals were allocated to family members doing heavy farm work rather than prioritizing the PLHIV and young children (Mangesho, 2011). Aging family members were also prioritized because of cultural practices of respect and kinship (Schatz, 2007; Schatz et al., 2011). Along with familial caregiving cultural expectations, household composition variations were essential factors in food choice and resource allocation among PLHIV households.

**Household wealth** refers to the financial capital and assets available within a household (26% of articles). Often, PLHIV families discussed the bi-directional relationship between food security and financial capacity to meet PLHIV needs (Chazan, 2014). Loss of livelihood and lack of remittances were the main economic shocks for the families as they juggled to meet the recommended diet and finances for HIV-related expenses (Moyo et al., 2017; King et al., 2018; Dovel and Thomson, 2016; Mill and Anarfi, 2002). Families discussed the socioeconomic barriers that reduced food consumption resources (Webel et al., 2017) and the competing cooking fuel costs for making special foods for the PLHIV (Beckett et al., 2016; Aga et al., 2009a; Ogunmefun and Schatz, 2009; Zembe et al., 2013). Families had to account for PLHIV's nutritional needs within the broader family budget (Table 3: 1.3) (Moyo et al., 2017). Additional wealth made hardships easier to handle as most families affected by HIV described a tremendous loss of labor of the PLHIV. In addition to food preparation and general care, the labor-intensive task of fetching water was described by PLHIV as furthering their dependency on others (Schatz, 2007). Their family caregiver and wealthier families could ease this burden by paying for care or labor assistance.

**Time use** refers to the time lost when PLHIV no longer participates in labor and household chores as well as the time that a family member spends on caring for the PLHIV (17% of articles). Time use negatively impacts household productivity (paid and unpaid) and well-being and affects food provisioning since family members use their time differently to ensure a PLHIV is cared for. In one study, participants observed that “the affected household may work daily, but the time is somehow shortened because they also have to care for the sick person” (Parker et al., 2009). The time use factor impacts varied by socio-economic status, and families affected by HIV described the heavy caregiving time burdens associated with providing special foods and the effects on daily routines and labor (Table 3: 1.4) (Parker et al., 2009; Kaler et al., 2010; Pallangyo and Mayers, 2009).

### 3.2 Characteristics refers to the composition that affects resources and decision-making regarding food preferences and how these factors affect PLHIV and family members’ food choices and consumption patterns. We found that food choices depended on family and/or household composition (generations, gender, aging), household health status (co-morbidities, chronic diseases), and household size

**Composition** refers to family members of different ages, generations, and genders residing in the same complex, whether under the same roof, within a shared compound, or in adjacent dwellings, influencing the dynamic of food choice. Generations refer to how multigenerational, extended, female- or male-headed households and children impacted food choices (42% of articles). An HIV diagnosis was often associated with a reshuffling that changed the dynamics within the composition. Older parents cared for their adult children with HIV as well as their young grandchildren. Recently widowed women impacted by HIV often moved to live with their parents for support with food and care (Table 3: 2.1a) (Nagata et al., 2012; Ssengonzi, 2007; Klunklin and Greenwood, 2005). Food consumption and choice were affected by age, marital status, and the number of children in the household (Table 3: 2.1b) (Nagata et al., 2012; Conroy et al., 2018; Weiser et al., 2010). A consistent cross-cutting theme was sharing food aid with children. Within multigenerational households, family food choices reflected a balancing act that aimed to meet the needs of children and the PLHIV (Klunklin and Greenwood, 2005).

Gender refers to how social roles ascribed to men and women impact food choices (36% of articles). The gender of the PLHIV influenced food allocation and choices within the household. The prioritization of the well-being and diets of PLHIV who are men focused on supporting them to recover and return to work, while PLHIV who are women received less family support (Kohli et al., 2012). The gender of the caregiver influenced the caregiving role and responsibilities for the household member with HIV. Women were considered the family's primary caregivers and food providers (Table 3: 2.2) (Davis and Kostick, 2018; Baylies, 2002), and thus served as caregivers for both the PLHIV in the family (Ssengonzi, 2007; Weiser et al., 2010; Seeley et al., 1993).

“Women and men both experienced significant food insecurity, but men were at times favored in terms of food distribution within the household. As explained by one HIV-positive widow: ‘Before you get married, your parents tell you that you're supposed to feed your husband, that he must eat more food. So when I got to my husband's home, whether I was sick or anything, he must have more food according to what I was told.’” (Weiser et al., 2010)

Williams and colleagues found that the impact of prioritization of men with HIV centered on meeting their immediate needs, so they consumed healthy diets (Williams and McGill, 2011). When the PLHIV was a woman, the focus was on long-term factors, family livelihoods, and psychological relief (Williams and McGill, 2011).

Aging refers to the increased health risks and additional support that PLHIV need later in life (8% of articles). In these articles, a common theme was that older people with HIV required more care, medications, food, and support for daily activities. They often had multiple co-morbidities, especially mental health, stigma, and a need for social support and financial security. As PLHIV age, there is a need for more significant support for daily living activities, such as cooking and fetching water (Table 3: 2.3a) (Wright et al., 2012). When an HIV diagnosis came later in life, aging PLHIV experienced stigma related to HIV and ageism (Araujo et al., 2018). Together, this dual stigma among the older PLHIV population led to high rates of non-disclosure and social withdrawal (Table 3: 2.3b) (Wright et al., 2012; Araujo et al., 2018).

**Household health status** refers to disease navigation or how the family and PLHIV make food decisions, comprised of co-morbidities of the PLHIV (12% of articles) and chronic diseases among other family members (7% of articles). PLHIV often have co-occurring morbidities, such as tuberculosis, hypertension, and diabetes, that require additional care and a tailored diet (Table 3: 2.4) (Webel et al., 2017). Families faced greater difficulty enabling PLHIV's food choices when other family members had chronic diseases. Family caregivers indicated high stress burdens affecting their mental well-being (Table 3: 2.5a-b) (Czaicki et al., 2017; Hatcher et al., 2020). Among both PLHIV and their family members, stressors associated with health and well-being made enhancing disease treatment through food access and choice more difficult (Table 3: 2.5c) (Pallangyo and Mayers, 2009).

**Household size** refers to the number of family members affecting the food choices of the PLHIV and household members (7% of articles). Themes focused on how PLHIV shared food aid with other household members (Table 3: 2.6) (Kalofonos, 2010), particularly children and neighbors, and how this pooling of resources meant there was less food available for the PLHIV (Schatz et al., 2011; Chazan, 2014; Dovel and Thomson, 2016; Aga et al., 2009a; Pallangyo and Mayers, 2009; Kalofonos, 2010; Rodas-Moya et al., 2016; Byron et al., 2008; Russell et al., 2016; Raniga and Simpson, 2010).

### 3.3 Action orientation refers to strategies and observable acts affecting food allocation decisions and diets of PLHIV. The strategies and acts are contingent on family support (or lack of it due to stigma) and value negotiations due to competing basic needs and family food preferences

Support refers to family factors that enable the food choices of PLHIV and their family members (68% of articles). The level of family support affects PLHIV food choices and acts along a continuum that varies over time. Continuous support played a significant role in overcoming stigma and supporting food preferences of PLHIV (Aransiola et al., 2014; Xie et al., 2017), “My mother told me to treat myself; if I [want] special foods, to just buy and eat them” (Xie et al., 2017). In resource-constrained contexts, families could often only provide intermittent support, often unpredictable and unreliable based on livelihood opportunities. They described stepping in at times of greater need, such as greater disease severity (Table 3: 3.1a) (Paz-Soldán et al., 2013), through the provision of money and food from their adult children or extended family (Table 3: 3.1b) (Moyo et al., 2017; Derose et al., 2017). Others experienced non-existent support when family members did not provide any support or negatively impacted their well-being. Levels of family support are influenced by stigma, shame, discrimination, knowledge about HIV transmission, and socioeconomic status. Food was the primary medium through which family support, stigma/shame, and discrimination were visibly expressed (Table 3: 3.1c) (Kohli et al., 2012). Lack of family support resulting from shame and stigma was observed in multiple ways including the delay of food preparation, which negatively affected the taking of medication and adherence, not buying desired foods, not sharing utensils and plates, and restricting certain foods like meat or fatty foods, increasing food insecurity for PLHIV (Table 3: 3.1d) (Derose et al., 2017).

**Value negotiations** refer to factors that compete with individual preferences within the family, including competing basic needs and family preferences. Competing basic needs (e.g., water, school, electricity, rent) refers to prioritizing one family member over another when household resources are scarce, thereby impacting the well-being of family members (37% of articles). Families described how they negotiate housing (Table 3: 3.2a-b) (Pallangyo and Mayers, 2009; Mkandawire et al., 2015; Atukunda et al., 2017), education (Tuller et al., 2010), medical treatment (additional testing, transportation cost, fees) (Pallangyo and Mayers, 2009; Atukunda et al., 2017; Tuller et al., 2010), and food costs (Pallangyo and Mayers, 2009; Atukunda et al., 2017; Tuller et al., 2010), especially since nutritious foods were more expensive. As one mother of five children mentions, the difficult choices between various basic needs (Tuller et al., 2010):

“Yes, I think about that 20,000 [to pay for transportation], I think about the fact that if I didn’t have HIV, I wouldn’t have to spend that money to come here for treatment. I imagine all the other things it could have been used for, and I don’t feel peace in my heart. I could hire people to do the digging, pay for school fees, buy more food. There’s no way I can even think of eating chicken, fish and meat as often as I’d like when I have to get money for transport to this place.” (Tuller et al., 2010)

Families negotiated the value of each short-term need with the needs of PLHIV. Efforts were made to enable PLHIV food choices even when jeopardizing long-term food security and assets of the household (Table 3: 3.2c) (Kaler et al., 2010; Thomas, 2006).

Family desirability refers to balancing all family food preferences and needs while accounting for norms related to religion, ethnicity, culture, or region (4% of articles). In one study, religious norms and festivals, such as fasting, guided PLHIV meal frequency, and anti-retroviral treatment (ART) adherence (Table 3: 3.3) (Bezabhe et al., 2014).

### 3.4 Health context refers to the chronic disease of focus. We specifically evaluated how the FDF fits within the chronic disease nature of HIV. We found that HIV had a long-term impact on livelihoods with enormous healthcare demands, the family food environment required the family's acceptance of the disease, and their nutritional awareness affected food choice

**Impact on livelihoods** refers to the lost income due to disease management of an individual with a chronic disease and their family members (49% of articles). Loss of livelihood due to HIV affected family food security and food choice (Table 3: 4.1a) (Pallangyo and Mayers, 2009), especially male-headed households in two ways. First is the loss of wages from men serving as the primary breadwinners. Second, because fewer income-generating opportunities existed for women and many earned lower wages than men, women had to engage in multiple income-generating activities which added to their stress (Table 3: 4.1b-c) (Ogunmefun and Schatz, 2009; Parker et al., 2009; VanTyler and Sheilds, 2015). Lastly, ART adherence was challenged by PLHIV employment due to the frequency and duration of clinic visits and mid-day or timed food consumption (Table 3: 4.1d-e) (Gombachika et al., 2014).

**Healthcare** refers to the burden associated with disease treatment, including hidden costs such as clinic transportation, waiting time, and testing, and how these healthcare burdens impact food choices (40% of articles). Household resources had to accommodate clinic visits' impact on incomes/livelihoods (Parker et al., 2009; Atukunda et al., 2017). There are additional direct costs associated with transportation and food consumption while going to/from the clinic to get medications and laboratory tests. As families try to account for these costs, they also have income/livelihood loss from allocating time to travel for clinic visits. The cost of HIV treatment (transport, time off work, tests) affected the cash available for food, especially the purchase of nutritious food, which was prohibitively higher (Table 3: 4.2) (Tuller et al., 2010; Balcha et al., 2011).

Community support refers to the structural networks, hospitals, and organized community groups an individual with a chronic disease can rely on to support their food choices (38% of articles). PLHIV groups and clinics also allowed forming PLHIV groups where they relied on each other to access food (Table 3: 4.3) (Aransiola et al., 2014; Horn and Brysiewicz, 2014; Hussen et al., 2014). Housing insecurity was commonly associated with losing livelihood, HIV-associated discrimination, and land grabbing from recently widowed women following the death of their husband who was affected by HIV (Schatz, 2007; Chazan, 2014; Beckett et al., 2016; Aga et al., 2009a; Parker et al., 2009; Mkandawire et al., 2015; Alomepe et al., 2016; Andersen, 2012; Burgess and Campbell, 2014; Du and Lekganyane, 2010; Kang'ethe, 2009a; Olenja, 1999; Schatz and Gilbert, 2012). Aga and colleagues explained, “Due to stigma and discrimination, these family caregivers faced difficulties in finding rental houses and in using communal facilities, like latrines and kitchens” (Aga et al., 2009a).

**Acceptance** refers to how the household's awareness of the chronic disease status and their demonstration of acceptance through the levels of support they provide (34% of articles). PLHIV often were reluctant to disclose their HIV status (Table 3: 4.4a), which was a key determinant of family support and food choices. Stigma had an erosive weathering effect on familial networks, support, and social capital (Aransiola et al., 2014). This stigma was also enacted within families who expected PLHIV to use separate eating utensils (Table 3: 4.4b-d) (Derose et al., 2017; Jones et al., 2009). Conversely, several articles identified sharing utensils, plates, drinking water, and food as a way to positively express support (Kohli et al., 2012; Aransiola et al., 2014; Wacharasin and Homchampa, 2008; Ngamvithayapong-Yanai et al., 2005; Miller and Tsoka, 2012; Li et al., 2008).

**Nutritional awareness** refers to how the family domain works to optimize the personal food environment by enabling healthier food choices for an individual with a chronic disease when family members know the person's nutritional needs (15% of articles). Disclosure of HIV status to family members was associated with greater awareness of the importance of nutrition and influenced both family and PLHIV's food choices. Targeted HIV-nutrition education to households raised awareness of PLHIV dietary needs, including scheduled eating around ART (Aga et al., 2009b), and were key to optimal outcomes among PLHIV. Family nutrition knowledge positively impacted PLHIV nutrition as family members cooked special meals, encouraged eating more fruits and vegetables, and avoided raw foods and alcohol (Table 3: 4.5a) (Wacharasin and Homchampa, 2008). Nutrition knowledge did not always translate to consumption behaviors, given that many families face severe financial constraints, loss of livelihood, and competing demands (Table 3: 4.5b-c) (Thomas, 2006; Laker and Ssekiboobo, 2003). Perceptions of healthy food vary with socioeconomic status, with those with low income focused on adequate food quantity. Support can occur at the expense of the health of family members as they forgo food consumption to meet the dietary needs of PLHIV (Gwatirisa and Manderson, 2009), but wealthier households could focus on culturally desirable food and diverse diets with reduced fat and alcohol.

## 4 Discussion

Family both enables and bounds agency in food consumption and plays a vital role in food access, food choice, and mitigation of health outcomes (Delormier et al., 2009, Giddens, 1991, Slater et al., 2012). In this review, we used Giddens' theory to inform the expansion of Turner's food environment framework to include the family food environment domain among families affected by HIV in LMICs (Turner et al., 2020; Giddens, 1991; Slater et al., 2012). Using qualitative evidence synthesis with a best-fit framework approach, we expanded the LMIC food environment framework within the context of families affected by HIV to develop the Family Dynamics Food Environment Framework (FDF). The 138 qualitative articles identified three major inter-connected domains under FDF through which family decision-making occurs on food choice: resources, characteristics, and action orientation, with the context of a health disease. Within these domains, most research has focused on how family food choices are affected by family support, livelihoods, social capital, and household composition. Other critical dimensions include competing basic needs, costs associated with disease treatment, and resource allocation. The family food environment domain interacts with and represents the complex dynamic of various domains and dimensions, influencing how PLHIV acquire and consume food. The interrelationships of family characteristics were found with livelihoods, social capital, competing basic needs, and gender roles affecting family food choices. Social capital intersected with the type of support PLHIV received and offset costs associated with disease treatment. Gender roles commonly intersect with family composition and social capital.

Many frameworks address family components for HIV care and treatment. Weiser and colleagues seminal work presented the bidirectional relationship between food insecurity and HIV infection, highlighting the role of household dynamics (Weiser et al., 2011). The family caregivers’ conceptions of the care model by Aga and colleagues identified themes that address the food-health needs of PLHIV, mainly symbolic gestures by family members to maintain routine, normalcy, and acceptance despite deprived economic conditions (Aga et al., 2009b, 2014). In the model of interrelationships between HIV, labor, and livelihoods, Parker and colleagues identified how family members (male, female, children) labor changed with different stages of HIV infections, ultimately affecting farming decisions and food security (Parker et al., 2009). Karney et al. and Conroy et al. applied dyadic interdependence theory to an HIV context, offering insights into how marital relationships affect household food security, health-seeking behaviors, and treatment adherence, especially on the role of gender and power to enable or constrain these relationships between couples (Conroy et al., 2018; Karney et al., 2010). A review of barriers to HIV care in East Africa identified family support as critical in realizing care and how stigma and its consequences are gendered (Ayieko et al., 2018). A qualitative meta-synthesis among pregnant women affected by HIV found family stigma a critical aspect of care because “living with people who have HIV requires that people in the environment learn adaptive behaviors and new knowledge to protect and assist these individuals” (Leyva-Moral et al., 2017). Lastly, Iwelunmor and colleagues use the PEN-3 cultural model to highlight families' role in stress, stigma, support, decision-making, and management of PLHIV care (Iwelunmor et al., 2008). These articles highlight the immediate and critical role of the family unit in addressing dietary, social, economic, emotional, and health-seeking aspects of HIV treatment and care. The Family Dynamics Food Environment Framework (FDF) developed here adds a valuable component of family as a social and economic unit for food choice and nutrition in the context of chronic disease.

Our study illuminates the various ways that household food dynamics, the health status of household members, and food choices, interact to ultimately affect decision-making processes for food consumption in the context of chronic disease management in low-resource settings (Messer, 1997). In related work, Lee and colleagues examined food choices since a tuberculosis diagnosis in Peru. They found dietary shifts towards “traditional” foods, with family members as the primary source of knowledge and support (Lee et al., 2020). Similarly, Perez-Leon and colleagues found the family accommodated their family member with type 2 diabetes and hypertension by adopting new dietary habits or minimal cooking methods (e.g., less salt or spices, removing portions of the food) to maintain single cooking preparations rather than multiple meals catering to individual dietary needs (Perez-Leon et al., 2018). We found elements of the FDF similar to other intra-household allocation of food and health frameworks (Messer, 1997; Harris-Fry et al., 2017). In a review of food allocation in Southeast Asia, Harris-Fry and colleagues identified household-level factors as key determinants of food allocation: food insecurity, scarcity, household income, education, nutrition knowledge, size, structure, religion, and ethnicity (Harris-Fry et al., 2017). The recent movement towards understanding food choice across a variety of contexts and themes (e.g., food safety, intergenerational food choices) helps us operationalize the interaction between external and internal food environments (Boncyk et al., 2022; Drew et al., 2022; Isanovic et al., 2023; Reyes et al., 2021; Samaddar et al., 2020; Schreinemachers et al., 2021; Wertheim-Heck and Raneri, 2019; Downs et al., 2022; Karanja et al., 2022; Flax et al., 2020; Green et al., 2020; Bukachi et al., 2021; Nordhagen et al., 2022). Lastly, the FDF has overlapping dimensions with previous high-income countries' food choice frameworks, such as occupation, time, gender roles, and value negotiations among families with school-aged children in Canada (Slater et al., 2012) and middle-income families from New York, USA (Furst et al., 1996). This overlap suggests some food choice dimensions are globalized, likely because of the globalized concept of work and school schedules (e.g., 9-to-5 work schedules).

Our analysis used a theory-driven approach with an *a priori* framework guided by Gidden's structuration theory and Turner's food environment framework (Turner et al., 2020; Turner et al., 2018; Giddens, 1991; Anthony, 1984). In addition to a systematic approach, we included gray literature and identified records through references. However, this review does have limitations. First, most included articles (>85%) were published before 2016. As families deal with ART adherence, additional factors might affect food choice as the HIV populations age, especially when dealing with mental health challenges and multiple NCD co-morbidities might become prominent (Patel et al., 2018; Kiplagat et al., 2022). Second, very few articles compare families and individual perspectives of the family food environment. Even in articles that interviewed the family members and PLHIV, limitations existed as virtually no study interviewed all family members. Third, children's food choices are important in the family food environment (Wertheim-Heck and Raneri, 2019). This review does not expand on children's food choices in PLHIV households. Lastly, most included articles were conducted in low-income populations. Variations in the interconnected dimensions from wealthier families in LMIC remain understudied. Further validation of the FDF within various families across all SES is warranted.

Poor diet is one of the leading causes of mortality worldwide (Afshin et al., 2019). As food environments rapidly shift towards ultra-processed, energy-dense foods in Southern and Eastern Africa and Asia, where many families affected by HIV live, there is an increased risk for diet-related NCDs among PLHIV and family members who are not living with HIV. Family is an essential intermediary between the external and internal food environments that can enable or bind food choice and operationalize social, economic, and personal factors related to food choice. With rapidly shifting food environments towards cheap, unhealthy foods, intra-household decision-making on food and managing health conditions will play a more significant role in the family food environment (Messer, 1997; Grey et al., 2015). Here, we examined the family food environment in the context of health and illness, which will become an essential integration in nutrition policies as NCD burdens grow in LMICs (Messer, 1997). The resource allocation towards health expenditure affects resource allocation to healthy food choices as families deal with costs associated with increasing morbidities. The FDF presented here, in the context of families affected by HIV, could be readily transferred and generalizable for other chronic and diet-related diseases. FDF could guide intervention design and nutritional policies that are effective and optimal for the entire family.

## Authorship

RA conceptualized the study aim and design. RA and MB performed data extraction. RA, MP, and CLP led the analysis, wrote the manuscript, and are primary responsibility for the final content. All authors provided input on the manuscript, read, and approved the final manuscript. RA and MB are joint co-first authors.

## Funding sources

This research has been funded by the Drivers of Food Choice Competitive Grants Programs, funded by the UK Government's Foreign, Commonwealth & Development Office, and the Bill & Melinda Gates Foundation [ID: OPP1110043], and managed by the University of South Carolina, Arnold School of Public Health, USA.

## CRediT authorship contribution statement

**Ramya Ambikapathi:** Writing – review & editing, Writing – original draft, Visualization, Validation, Supervision, Resources, Project administration, Methodology, Investigation, Funding acquisition, Formal analysis, Data curation, Conceptualization. **Morgan Boncyk:** Writing – review & editing, Writing – original draft, Validation, Software, Project administration, Methodology, Formal analysis. **Nilupa S. Gunaratna:** Writing – review & editing, Supervision. **Wafaie Fawzi:** Writing – review & editing, Supervision. **Germana Leyna:** Writing – review & editing. **Suneetha Kadiyala:** Writing – review & editing. **Crystal L. Patil:** Writing – review & editing, Writing – original draft, Validation, Supervision, Project administration, Methodology, Funding acquisition, Formal analysis.

## Declaration of competing interest

The authors declare that they have no known competing financial interests or personal relationships that could have appeared to influence the work reported in this paper.

## Data Availability

No data was used for the research described in the article.
