# Supplementary material for: Expanding the food environment framework to include family dynamics: A systematic synthesis of qualitative evidence using HIV as a case study
Source: Glob Food Sec. 2024 Sep;42:100788. doi: 10.1016/j.gfs.2024.100788 (PMC11413529; doi:10.1016/j.gfs.2024.100788)
Supplement: Multimedia component 1 [file mmc1.docx]

Supplemental Figure 1: Map of included articles


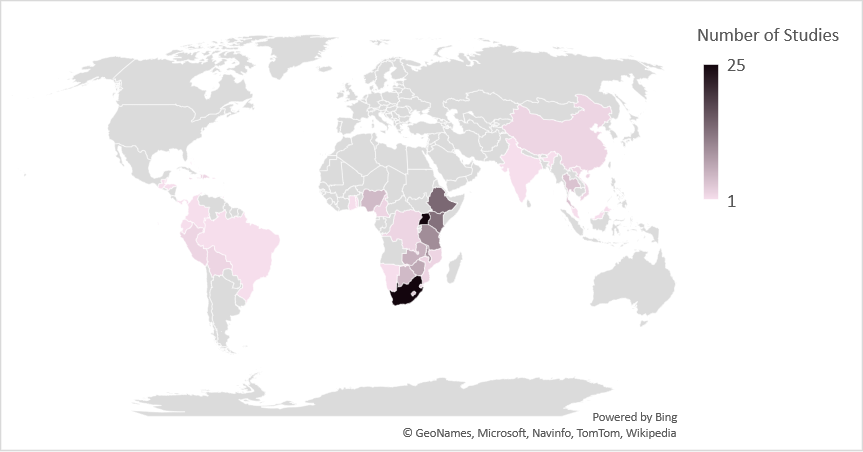

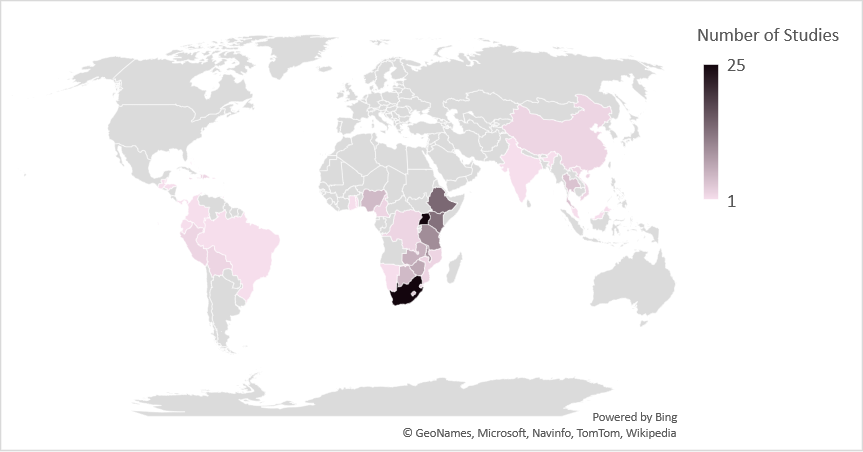


Number of articles

1

25

Supplemental Table 1. Quality of articles included in the HIV family food environment review (n=138) using a modified Critical Appraisals Skills Programme checklist.

| **Study quality** | **No** | **Yes** | |
| --- | --- | --- | --- |
|  | n | n | (%) |
| Are the results valid? |  |  |  |
| Was there a clear statement of the aims of the research? | 6 | 132 | 96% |
| Is a qualitative methodology appropriate? | 3 | 135 | 98% |
| Is it worth continuing? |  |  |  |
| Was the research design appropriate to address the aims of the research? | 19 | 119 | 86% |
| Was the recruitment strategy appropriate to the aims of the research? | 11 | 127 | 92% |
| Was the data collected in a way that addressed the research issue? | 3 | 135 | 98% |
| Has the relationship between researcher and participants been adequately considered? | 26 | 112 | 81% |
| What are the results? |  |  |  |
| Have ethical issues been taken into consideration? | 44 | 94 | 68% |
| Was the data analysis sufficiently rigorous? | 16 | 122 | 88% |
| Is there a clear statement of findings? | 3 | 135 | 98% |
